# Bmi1 Essentially Mediates Podocalyxin-Enhanced Cisplatin Chemoresistance in Oral Tongue Squamous Cell Carcinoma

**DOI:** 10.1371/journal.pone.0123208

**Published:** 2015-04-27

**Authors:** Yueying Zhou, Leiyi Zhang, Hao Pan, Baisheng Wang, Fei Yan, Xiaodan Fang, Krishna Munnee, Zhangui Tang

**Affiliations:** 1 Department of Stomatology, Xiangya Hospital, Central South University, Changsha, China; 2 Department of Minimal Invasive Surgery, The Second Xiangya Hospital, Central South University, Changsha, China; 3 Department of Maxillofacial Surgery, Xiangya Stomatological Hospital, Central South University, Changsha, China; 4 Department of Prosthodontics, Xiangya Stomatological Hospital, Central South University, Changsha, China; Universidade de São Paulo, BRAZIL

## Abstract

Oral tongue squamous cell carcinoma (OTSCC) is one of the most common head and neck cancers. Innate or acquired resistance to cisplatin, a standard chemotherapy agent for OTSCC, is common in patients with OTSCC. Understanding the molecular basis for cisplatin chemoresistance in OTSCC cells may serve as a basis for identification of novel therapeutic targets. Podocalyxin (PODXL) has been found critical for malignant progression in a variety of cancers. Bmi1 has recently been found to induce cell apoptosis and cisplatin chemosensitivity in OTSCC cells. In this study, we explored the interaction between PODXL and Bmi1 in OTSCC cells, and assessed its impact on OTSCC cell chemoresistance to cisplatin. PODXL and/or Bmi1 were stably overexpressed or knocked down in SCC-4 and Tca8113 human OTSCC cells. Overexpression of PODXL in both cell lines markedly elevated the expression level of Bmi1 and the half maximal inhibitory concentration (IC50) of cisplain and reduced cisplatin-induced cell apoptosis, which was abolished by knockdown of Bmi1 or a selective focal adhesion kinase (FAK) inhibitor. On the other hand, knockdown of PODXL significantly decreased the Bmi1 expression level and cisplatin IC50 and increased cisplatin-induced cell apoptosis, which was completely reversed by overexpression of Bmi1. While overexpression and knockdown of PODXL respectively increased and decreased the FAK activity, Bmi1 showed no significant effect on the FAK activity in OTSCC cells. In addition, overexpression of PODXL markedly elevated the stability of Bmi1 mRNA, which was abolished by a selective FAK inhibitor. In conclusion, this study provides the first evidence that PODXL up-regulates the expression level of Bmi1 in OTSCC cells by increasing the stability of Bmi1 mRNA through a FAK-dependent mechanism; this effect leads to enhanced cisplatin chemoresistance in OTSCC cells. This study adds new insights into the molecular mechanisms underlying OTSCC chemoresistance.

## Introduction

Oral squamous cell carcinoma is a lethal disease estimated to have a 275,000 incidence per year [1]. It accounts for more than 90% of all head and neck cancers and has a poor prognosis [2]. Tongue cancer is the most common oral cancer [1]. The development of oral tongue squamous cell carcinoma (OTSCC) metastasis poses clinical challenges because of the limited therapeutic options available [3]. Despite great advances in multimodal therapies against OTSCC over the past decades, the overall 5-year survival rate with this disease has not been markedly improved [4]. The standard of care for OTSCC used to be surgery and radiation. The addition of platinum-based drugs led to an improvement in local disease control and organ preservation [5]. Cisplatin, one of the most potent platinum-based chemotherapeutic agents currently in use, is effective as a single agent or in combination with other drugs for the treatment of OTSCC [6]. Treatment with cisplatin-based chemotherapy has been found to improve the prognosis of patients with OTSCC [7]. However, one of the most important clinical problems for cisplatin-based OTSCC chemotherapy is the intrinsic/acquired chemoresistance to cisplatin [8].

B lymphoma Mo-MLV insertion region 1 homolog (Bmi1) is a member of the polycomb repressive complex 1 (PRC1) that functions as an epigenetic silencer of many target genes such as Ink4a-arf locus [9]. Accumulating evidence has shown that aberrant overexpression of Bmi1 is correlated with advanced stages, aggressive clinicopathological behavior, therapeutic resistance and poor prognosis in myeloid leukemia, lung cancer, colorectal cancer, and head and neck cancer [10]. A recent study showed that Bmi1 knockdown inhibited cell proliferation and migration, induced cell apoptosis and senescence, and enhanced cisplatin chemosensitivity in OTSCC cells [10], indicating that Bmi1 serves as a key driver and biomarker with multiple oncogenic functions underlying tongue tumorigenesis.

Podocalyxin (PODXL) is an anti-adhesive transmembrane sialomucin normally expressed on the free, unattached surface of hematopoietic progenitors and megakaryocytes [11]. Recent studies have shown that PODXL is also expressed in a variety of cancers [11–18]. The clinical significance of PODXL in cancer progression has been investigated in numerous tumor types, including breast, colon, and uterine carcinoma [11]. It has been found that overexpression of PODXL is associated with increased aggressiveness of breast and prostate cancer cells [16, 17].

Our pilot study suggested that PODXL could regulate the expression level of Bmi1 in OTSCC cells. In this study, we explored the interaction between PODXL and Bmi1 in OTSCC cells, and assessed its impact on OTSCC cell chemoresistance to cisplatin.

## Materials and Methods

### Transfection and lentiviral transduction

Human OTSCC cell lines SCC-4 and Tca8113 were purchased from the American Tissue Culture Collection (ATCC, Manassas, VA, USA) and Wuhan Boster Bio-Engineering Inc. (Wuhan, PR China), respectively. Human full length PODXL cDNA was subcloned into the pcDNA 3.1 plasmid. Human Bmi1 cDNA clone (SC116894) was purchased from Origene (Beijing, China) and the full length Bmi1 cDNA sequence was subcloned into the pcDNA 3.1 plasmid. The PODXL and the Bmi1 expression vectors were respectively transfected into cells using Lipofectamine 2000 transfection reagent (Life Technologies, Carlsbad, CA, USA) according to the manufacturer’s instructions. Pools of stable transductants were generated via selection with G418 (700 μg/ml; Sigma-Aldrich, Beijing, China) by the manufacturer’s protocol. The PODXL PODXL (sc-44029-V) and Bmi1 (sc-29814-V) shRNA lentiviral particles purchd from Santa Cruz Biotechnology (Santa Cruz, CA, USA) contain expression constructs encoding target-specific shRNA designed to specifically knockdown PODXL and Bmi1 gene expression, respectively. The control shRNA lentiviral particles (sc-108080; Santa Cruz Biotechnology) contain a scrambled shRNA sequence that will not lead to specific degradation of any cellular mRNA. Lentiviral transduction was performed and pools of stable transductants were generated via selection with puromycin (5 μg/mL; Sigma-Aldrich) by the manufacturer’s protocol (Santa Cruz Biotechnology).

### Western blot analysis

SCC-4 and Tca8113 cells was lysed with a hypotonic buffer containing 2% Nonidet-P and a protease inhibitor cocktail (Sigma-Aldrich) by sonication three times for 3 seconds on ice. The supernatant obtained after centrifugation at 2000 *g* for 15 miutes at 4°C was used for protein concentration determination by the Coomassie blue method and for subsequent steps. Equal amount of proteins for each sample were separated by 10% SDS-polyacrylamide gel and blotted onto a polyvinylidene difluoride microporous membrane (Millipore, Billerica, MA, USA). Membranes were incubated for 1 hour with a 1:1000 dilution of mouse monoclonal anti-PODXL (3D3) (39–3800) antibody (Life Technologies), rabbit polyclonal anti-Bmi1 (H-99; sc-10745) antibody (Santa Cruz Biotechnology) or mouse monoclonal anti-glyceraldehyde-3-phosphate dehydrogenase (GAPDH) (6C5; sc-32233) antibody (Santa Cruz Biotechnology), and then washed and revealed using secondary antibodies with horseradish peroxidase conjugate (1:5000, 1 hour). Peroxidase was revealed with a GE Healthcare ECL kit (Shanghai, China). Three independent experiments were performed.

### Real-time quantitative reverse transcription PCR

RNA was prepared from cells using TRIzol reagent. The cDNAs were synthesized using SuperScript II reverse transcriptase (Life Technologies). Real-time quantitative PCR was performed on an Abi-Prism 7700 Sequence Detection System, with use of the fluorescent dye SYBR Green Master Mix (Applied Biosystems, Beijing, China) as described by the manufacturer. The primers used are as follows: for human Bmi1, 5′-TCATCCTTCTGCTGATGCTG-3′ (forward) and 5′-CCGATCCAATCTGTTCTGGT-3′ (reverse); for human GAPDH, 5′-GACTCATGACCACAGTCCATGC-3′ (forward) and 5′-AGAGGCAGGGATGATGTTCTG-3′ (reverse). Relative quantification of the mRNA level of Bmi1 was determined using the 2^-ΔΔCt^ method and normalized against that of GAPDH in the same sample [19]. Each experiment was repeated for three times in duplicates.

### Luciferase Assay

Cells were transfected with a commercially available human Bmi1 promoter/luciferase reporter plasmid (S711041; SwitchGear Genomics, Shanghai, China) using Lipofectamine 2000 transfection reagent (Life Technologies) and then cultured for 24 hours. Luciferase assays were performed with the Dual-Luciferase Reporter Assay system (Promega, Madison, WI, USA) according to the manufacturer’s instructions. Each experiment was repeated for three times in duplicates.

### mRNA stability assays

Two assays were performed to determine the stability of Bmi1 mRNA as follows: (1) SCC-4 and Tca8113 cells were pre-treated with transcription inhibitor actinomycin D (1 mg/mL) (Sigma-Aldrich) for 30 minutes, and then cultured for 1, 2 or 4 hours in culture medium containing actinomycin D (1 mg/mL). Then the mRNA level of Bmi1 was determined with real-time quantitative reverse transcription PCR as described above. (2) A Click-iT Nascent RNA Capture Kit (C-10365; Life Technologies) was used to determine the stability of Bmi1 mRNA according to the manufacturer’s instructions. Briefly, SCC-4 and Tca8113 cells were labeled with 0.2 mM ethynyl uridine (EU) and incubated at 37°C for 4 hours. Cells were then allowed to recover in EU-free medium for 0, 1, 2 or 4 hours, respectively. Total RNA was extracted and 5 μg of total RNA was mixed with Click-iT reaction cocktail (25 μL Click-iT EU buffer, 4 μL 25mM CuSO4 and 2.5 μL 10mM Biotin azide). Immediately, the reaction buffer additive 1 was added, following by reaction buffer additive 2 exactly 3 minutes after adding of the first additive, and the reaction was carried out for 30 minutes at room temperature. Following incubation, the ‘clicked’ RNA was re-purified by ammonium acetate precipitation, and 0.5 μg of purified RNA was bound to 25 μL of streptavidin magnetic beads with 80 units of RNAseOUT Recombinant Ribonuclease Inhibitors (Life Technologies) for 30 minutes. Beads were then washed 5×300 μL of Click-iT wash buffer 1, followed by 5×300 μL of wash buffer 2, and re-suspended in a final volume of 25 μL wash buffer 2. The captured RNA was in-bead converted to cDNA as per manufacturer’s instructions using SuperScript III reverse transcriptase (Life Technologies). Then the mRNA level of Bmi1 was determined with real-time quantitative reverse transcription PCR as described above.

### Cisplatin chemosensitivity assay

Cells were plated in 96-well plates at a density of 2000 cells. After 24 hours of incubation, the medium was replaced by fresh medium with or without various concentrations of cisplatin (Sigma-Aldrich). Then cell viability was assayed 48 hours later using a modified MTT assay as previously described [20]. The half maximal inhibitory concentration (IC50) was defined as the concentration resulting in a 50% reduction in growth compared to control cell growth. Each experiment was repeated for three times in duplicates. The IC50 dose-response curves were plotted with GraphPad Prism Version 5.0 (GraphPad Software, La Jolla, CA, USA).

### Cell apoptosis assay

Cells were cultured at 9 × 10^4^ cells per well in 96-well tissue culture plates and incubated at 37°C for 12 or 24 hours under cisplatin (10 μM) treatment. Cell apoptosis was measured at 12 and 24 hours with a microplate reader-based TiterTACS in situ apoptosis detection kit (4822-96-K; R&D systems, Minneapolis, MN, USA) as described by the manufacturer [21]. Each experiment was repeated for three times in duplicates.

### FAK activity assay

Kinase activity of FAK was measured with a nonradioactive isotope solid-phase enzyme-linked immunosorbent assay (ELISA) kit using the poly(Glu, Tyr) as a substrate (Universal Tyrosine Kinase Assay kit; Takara Biomedical Technology, Beijing, hina). FAK was purified from cells by immunoprecipitation with a mouse monoclonal anti-focal adhesion kinase (FAK) (B-8; sc-271195) antibody (Santa Cruz Biotechnology). Immunoprecipitates were subjected to the in vitro kinase assay as per the manufacturer’s instructions (Takara Biomedical Technology). Each experiment was repeated for three times in duplicates.

### Statistical analysis

Statistical analyses were performed with SPSS for Windows 10.0 (SPSS Inc., Chicago, IL, USA). All data values were expressed as means±SD. Comparisons of means among multiple groups were performed with one-way ANOVA followed by *post hoc* pairwise comparisons using Tukey’s tests. A two-tailed *p*<0.05 was considered statistically significant in this study.

## Results

### PODXL up-regulates the expression level of Bmi1 in OTSCC cells

**Fig 1:**
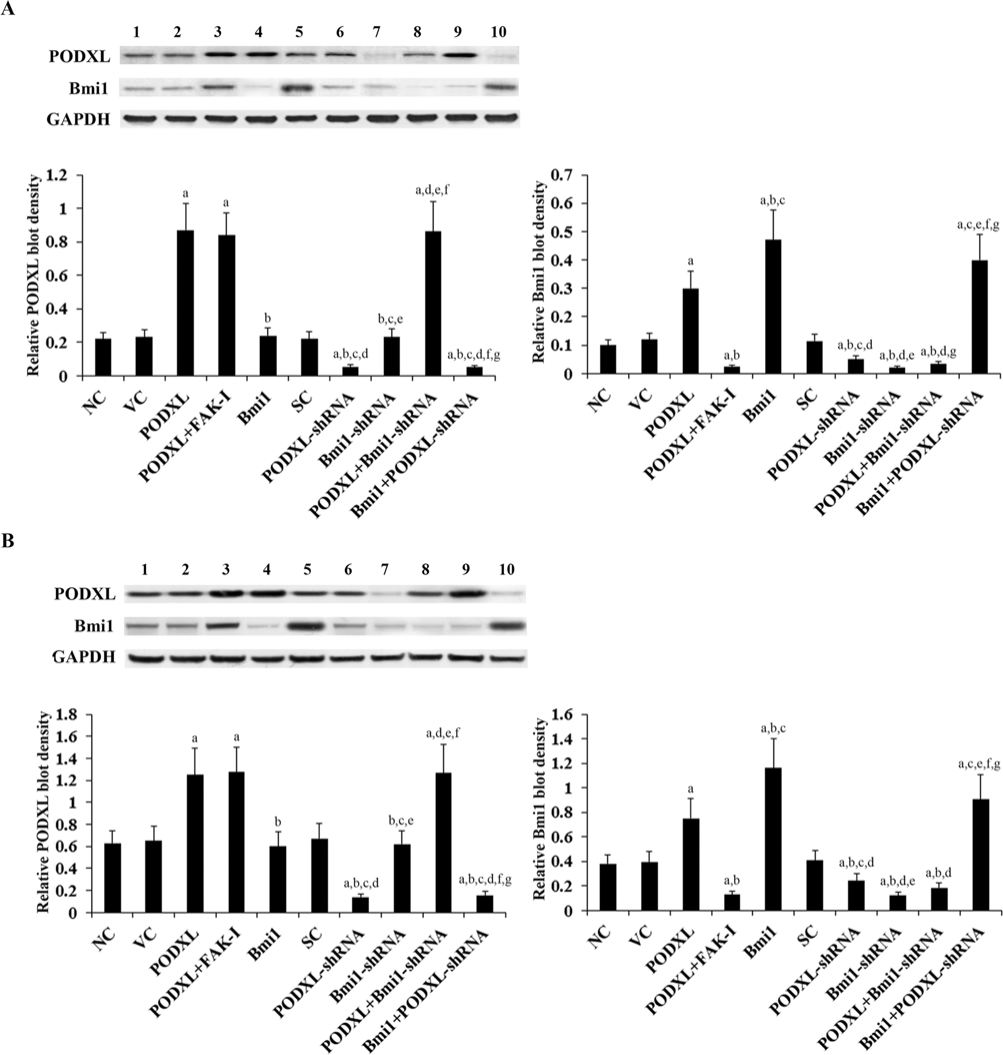
Protein levels of podocalyxin (PODXL) and Bmi1 in oral tongue squamous cell carcinoma (OTSCC) cells with overexpression and knockdown of PODXL and/or Bmi1. In (A) SCC-4 and (B) Tca8113 OTSCC cells, the protein levels of PODXL and BMI1 were determined with western blot analysis in normal control cells (NC, lane 1), cells stably transfected with the empty pcDNA 3.1 vector (VC, lane 2), cells stably transfected with PODXL (lane 3), cells stably transfected with PODXL and treated with focal adhesion kinase (FAK) inhibitor 14 (50 M) for 24 hours (PODXL+FAK-I, lane 4), cells stably transfected with Bmi1 (lane 5), cells stably transduced with scramble control shRNA (SC, lane 6), cells stably transduced with PODXL-shRNA (lane 7), cells stably transduced with BMI1-shRNA (lane 8), cells stably transfected with PODXL and transduced with BMI1-shRNA (PODXL+BMI1-shRNA, lane 9), and cells stably transfected with Bmi1 and transduced with PODXL-shRNA (Bmi1+PODXL-shRNA, lane 10). Glyceraldehyde-3-phosphate dehydrogenase (GAPDH) blotting was used as a loading control. Density of the Western blots was measured by densitometry, and the density of the PODXL and the Bmi1 blots was normalized against that of the GAPHD blot in the same sample to obtain a relative blot density to represent relative PODXL and Bmi1 content in each sample, respectively. ^a^ *p*<0.05 vs. controls (NC, VC and SC); ^b^ *p*<0.05 vs. PODXL; ^c^ *p*<0.05 vs. PODXL+FAK-I; ^d^ *p*<0.05 vs. Bmi1; ^e^ *p*<0.05 vs. PODXL-shRNA; ^f^ *p*<0.05 vs. Bmi1-shRNA; ^g^ *p*<0.05 vs. PODXL+Bmi1-shRNA.

**Fig 2:**
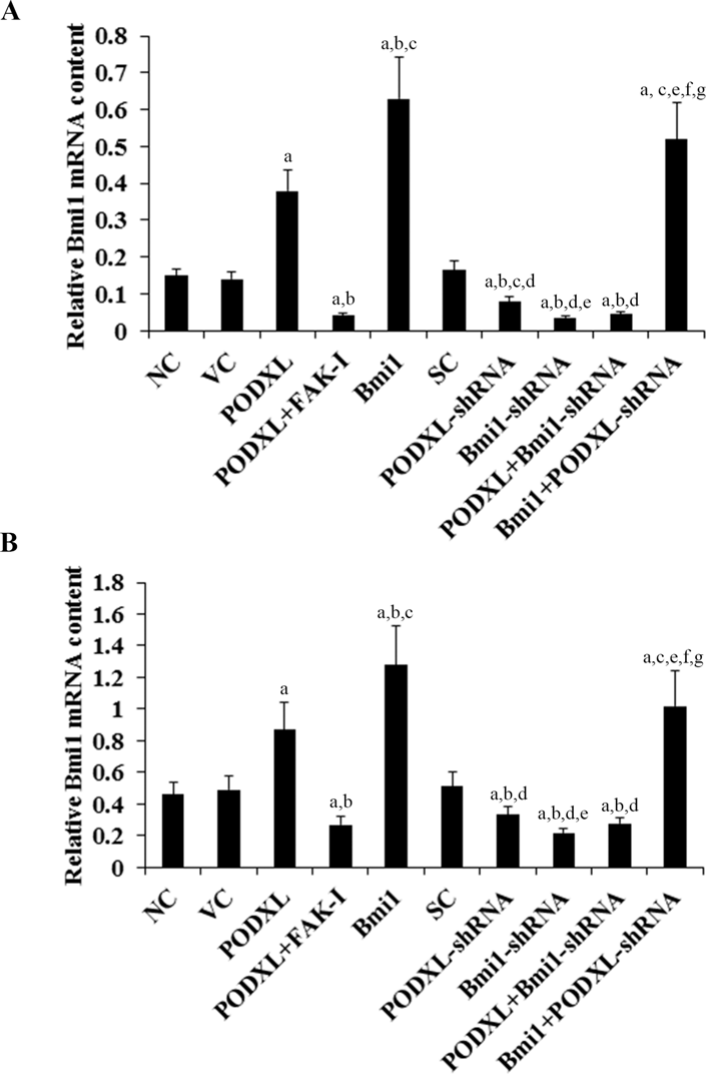
Effect of podocalyxin (PODXL) on the mRNA level of Bmi1 in oral tongue squamous cell carcinoma (OTSCC) cells. In (A) SCC-4 and (B) Tca8113 OTSCC cells, mRNA levels of Bmi1 were determined with real-time RT-PCR in normal control cells (NC), cells stably transfected with the empty pcDNA 3.1 vector (VC), cells stably transfected with PODXL, cells stably transfected with PODXL and treated with focal adhesion kinase (FAK) inhibitor 14 (50 M) for 24 hours (PODXL+FAK-I), cells stably transfected with Bmi1, cells stably transduced with scramble control shRNA (SC), cells stably transduced with PODXL-shRNA, cells stably transduced with BMI1-shRNA, cells stably transfected with PODXL and transduced with BMI1-shRNA (PODXL+BMI1-shRNA), and cells stably transfected with Bmi1 and transduced with PODXL-shRNA (Bmi1+PODXL-shRNA). ^a^ *p*<0.05 vs. controls (NC, VC and SC); ^b^ *p*<0.05 vs. PODXL; ^c^ *p*<0.05 vs. PODXL+FAK-I; ^d^ *p*<0.05 vs. Bmi1; ^e^ *p*<0.05 vs. PODXL-shRNA; ^f^ *p*<0.05 vs. Bmi1-shRNA; ^g^ *p*<0.05 vs. PODXL+Bmi1-shRNA.

To investigate functional interaction between PODXL and Bmi1 in OTSCC cells, we stably overexpressed PODXL and Bmi1 in SCC-4 and Tca8113 human OTSCC cells by stable transfection, and on the other hand stably transduced the cells with lentiviral shRNAs to knock down PODXL and Bmi1, respectively. Western blot analyses were performed to determine the protein levels of PODXL and Bmi1 in the cells. Density of the Western blots was measured by densitometry, and the density of the PODXL and the Bmi1 blots was normalized against that of the GAPHD blot in the same sample to obtain a relative blot density to represent relative PODXL and Bmi1 content in each sample, respectively. As shown in Fig 1, PODXL and Bmi1 are constitutively expressed in both SCC-4 and Tca8113 cells. Compared with the controls, PODXL was overexpressed about 3.9 folds in SCC-4 and 2 folds in Tca8113 cells; on the other hand, the endogenous PODXL level was knocked down over 75% in both SCC-4 and Tca8113 cells. Overexpression of PODXL increased the protein level of Bmi1 by about 3 folds in SCC-4 cells and 2 folds in Tca8113 cells; the effect was completely abolished by a selective FAK inhibitor (FAK inhibitor 14) [22]. On the other hand, knockdown of PODXL decreased the protein level of Bmi1 by about 50% in SCC-4 cells and 40% in Tca8113 cells (Fig 1). Stable transfection of Bmi1 increased the Bmi1 protein level by about 4.7 folds in SCC-4 cells and 3 folds in Tca8113 cells, with no significant effect on the expression of PODXL (Fig 1). FAK inhibitor 14 showed no significant effect on the protein level of PODXL in both cell lines (Fig 2). Real-time RT-PCR assays showed similar data trend (Fig 2) as in Fig 1. The results suggest that PODXL up-regulates the expression of Bmi1 in OTSCC cells at the mRNA level through a FAK-dependent mechanism.

### PODXL increases the stability of Bmi1 mRNA in OTSCC cells

**Fig 3:**
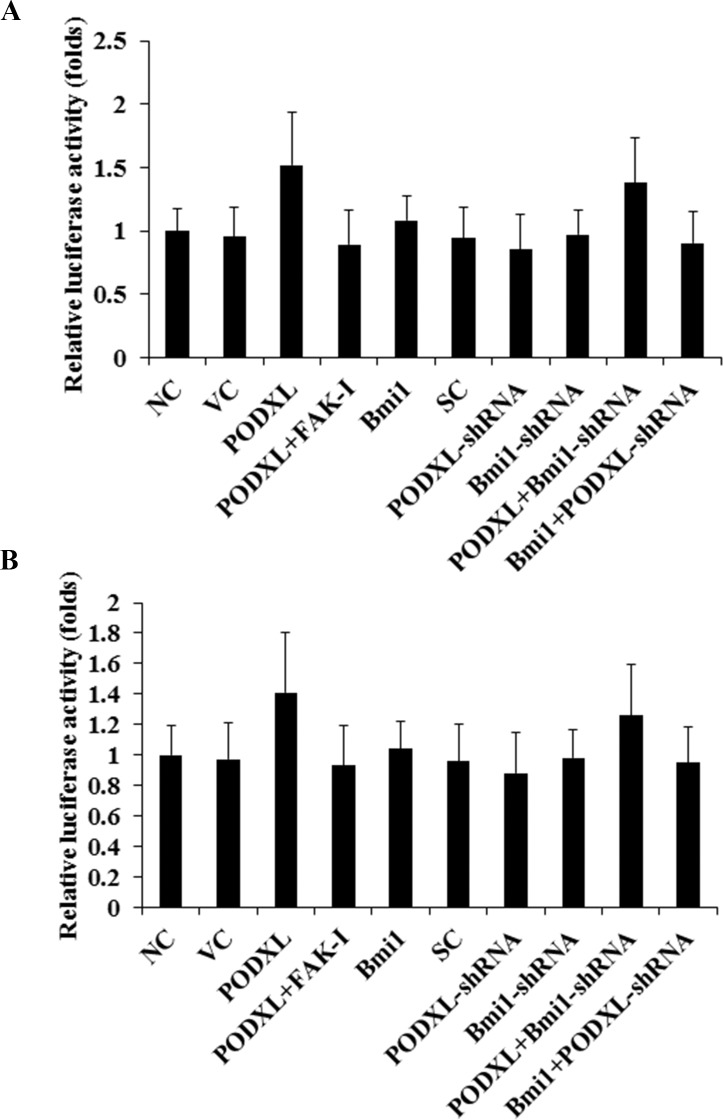
Effect of podocalyxin (PODXL) on Bmi1 promoter activities in oral tongue squamous cell carcinoma (OTSCC) cells. (A) SCC-4 and (B) Tca8113 OTSCC cells were transfected with human Bmi1 promoter/luciferase reporter plasmids and then cultured for 24 hours. Luciferase activities were determined in normal control cells (NC), cells stably transfected with the empty pcDNA 3.1 vector (VC), cells stably transfected with PODXL, cells stably transfected with PODXL and treated with focal adhesion kinase (FAK) inhibitor 14 (50 M) for 24 hours (PODXL+FAK-I), cells stably transfected with Bmi1, cells stably transduced with scramble control shRNA (SC), cells stably transduced with PODXL-shRNA, cells stably transduced with BMI1-shRNA, cells stably transfected with PODXL and transduced with BMI1-shRNA (PODXL+BMI1-shRNA), and cells stably transfected with Bmi1 and transduced with PODXL-shRNA (Bmi1+PODXL-shRNA). The luciferase activity was expressed as fold changes to that of NC (designated as 1).

**Fig 4:**
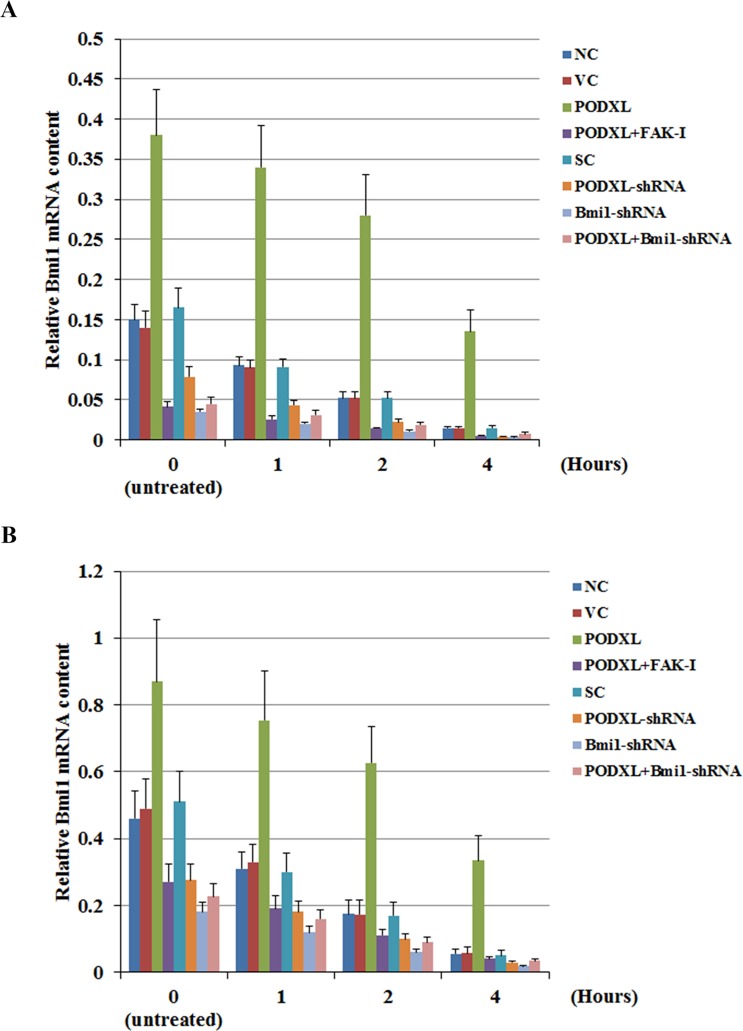
Effect of podocalyxin (PODXL) on Bmi1 mRNA stability in oral tongue squamous cell carcinoma (OTSCC) cells. (A) SCC-4 and (B) Tca8113 OTSCC cells were pre-treated with transcription inhibitor actinomycin D (1 mg/mL) for 30 minutes, and then cultured for 1, 2 or 4 hours in medium containing actinomycin D (1 mg/mL). The Bmi1 mRNA level were then determined with real-time RT-PCR in normal control cells (NC), cells stably transfected with the empty pcDNA 3.1 vector (VC), cells stably transfected with PODXL, cells stably transfected with PODXL and treated with focal adhesion kinase (FAK) inhibitor 14 (50 M) for 24 hours (PODXL+FAK-I), cells stably transfected with Bmi1, cells stably transduced with scramble control shRNA (SC), cells stably transduced with PODXL-shRNA, cells stably transduced with BMI1-shRNA, cells stably transfected with PODXL and transduced with BMI1-shRNA (PODXL+BMI1-shRNA), and cells stably transfected with Bmi1 and transduced with PODXL-shRNA (Bmi1+PODXL-shRNA).

To test whether PODXL up-regulated the Bmi1 mRNA level in OSTCC cells by transcriptionally activating the Bmi1 gene promoter, we transfected SCC-4 and Tca8113 cells with a human Bmi1 promoter/luciferase reporter. As shown in Fig 3, luciferase reporter assays revealed that overexpression and knockdown of PODXL had no significant effects on the Bmi1 promoter activity, suggesting that PODXL did not up-regulate the Bmi1 mRNA level in OSTCC cells at the gene promoter/transcription level. We next examined the effect of PODXL on Bmi1 mRNA stability. SCC-4 and Tca8113 cells were pre-treated with transcription inhibitor actinomycin D (1 mg/mL) for 30 minutes, and then cultured for 1, 2 or 4 hours in culture medium containing actinomycin D (1 mg/mL). The dose and duration of actinomycin D treatment were selected based on titration in SCC-4 cells (S1 Fig). As shown in Fig 4, after one hour of actinomycin D treatment, the Bmi1 mRNA level in the control cells (NC, VC, SC) dropped to about 65% of that in the untreated control cells; after four hours of treatment, the Bmi1 mRNA level in the control cells dropped to about 10% of that in the untreated control cells. In cells overexpressing PODXL, however, the Bmi1 mRNA level in the treated cells remained above 86% of that in the untreated cells after one hour of treatment; after four hours of treatment, the Bmi1 mRNA level in the treated cells remained above 36% of that in the untreated cells. This effect of PODXL was completely abolished by FAK inhibitor 14. On the other hand, in cells with knockdown of PODXL, the Bmi1 mRNA level in the treated cells dropped to about 55% of that in the untreated cells after one hour of treatment; after four hours of treatment, the Bmi1 mRNA level in the treated cells dropped to about 5% of that in the untreated cells. The results suggest that PODXL increases the stability of Bmi1 mRNA in OTSCC cells through a FAK-dependent post-transcriptional mechanism. To confirm the results, we further performed transcriptional pulse-chase assays using a Click-iT Nascent RNA Capture Kit (Life Technologies) (S2 Fig). Briefly, SCC-4 and Tca8113 cells were labeled with EU and incubated at 37°C for 4 hours. Cells were then allowed to recover in EU-free medium for 0, 1, 2 or 4 hours, respectively. Then the labeled RNA was captured and subject to real-time RT-PCR assays to determine the Bmi1 mRNA levels (S2 Fig), which showed similar data trend as in Fig 4.

### PODXL enhances cisplatin chemoresistance in OTSCC cells through Bmi1

**Fig 5:**
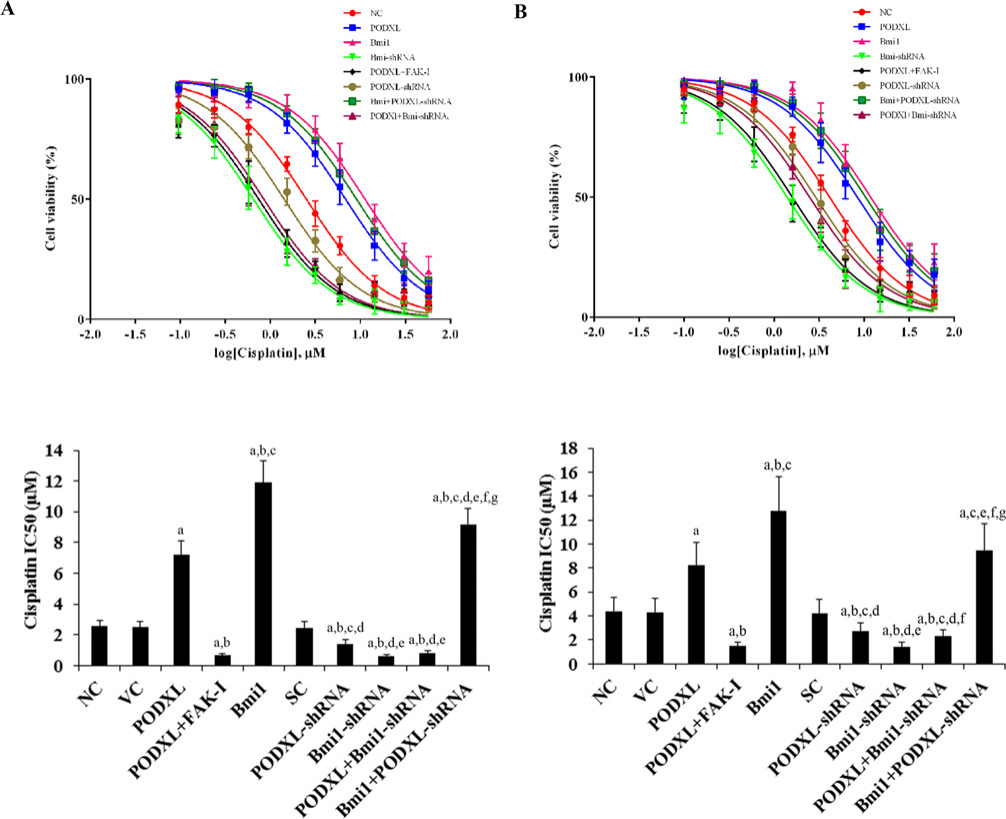
Effect of PODXL/Bmi1 signaling on cisplatin chemoresistance in oral tongue squamous cell carcinoma (OTSCC) cells. (A) SCC-4 and (B) Tca8113 OTSCC cells were treated with or without various concentrations of cisplatin for 48 hours. The half maximal inhibitory concentration (IC50) values were determined in normal control cells (NC), cells stably transfected with the empty pcDNA 3.1 vector (VC), cells stably transfected with PODXL, cells stably transfected with PODXL and treated with focal adhesion kinase (FAK) inhibitor 14 (50 M) for 24 hours (PODXL+FAK-I), cells stably transfected with Bmi1, cells stably transduced with scramble control shRNA (SC), cells stably transduced with PODXL-shRNA, cells stably transduced with BMI1-shRNA, cells stably transfected with PODXL and transduced with BMI1-shRNA (PODXL+BMI1-shRNA), and cells stably transfected with Bmi1 and transduced with PODXL-shRNA (Bmi1+PODXL-shRNA). The IC50 dose-response curves were plotted with GraphPad Prism 5.0 (GraphPad Software). The dose-response curves for VC and SC in both SCC-4 and Tca8113 cells are presented in S3 Fig, because they mostly overlap with the dose-response curve of NC. IC50 values (mean±SD) are presented by histograms. ^a^ *p*<0.05 vs. controls (NC, VC and SC); ^b^ *p*<0.05 vs. PODXL; ^c^ *p*<0.05 vs. PODXL+FAK-I; ^d^ *p*<0.05 vs. Bmi1; ^e^ *p*<0.05 vs. PODXL-shRNA; ^f^ *p*<0.05 vs. Bmi1-shRNA; ^g^ *p*<0.05 vs. PODXL+Bmi1-shRNA.

To explore the interaction between PODXL and Bmi1 on cisplatin chemoresistance in OTSCC cells, we examined cisplatin IC50 values in SCC-4 and Tca8113 cells. A higher IC50 value was considered to correspond to clinical chemoresistance to cisplatin. The IC50 dose-response curves were plotted with GraphPad Prism 5.0 (GraphPad Software) (Fig 5). The dose-response curves for pcDNA3.1 vector control (VC) and scramble shRNA control (SC) in both SCC-4 and Tca8113 cells are separately presented in S3 Fig, because they mostly overlapped with the dose-response curve of the NC control. As shown in Fig 5, for 48 hours of cisplatin treatment, the cisplatin IC50 values for SCC-4 and Tca8113 cells were 2.6 μM and 4.4 μM, respectively. Overexpression of PODXL significantly increased the IC50 values to 7.2 μM and 8.2 μM, respectively, which was abolished by FAK inhibitor 14 or knockdown of Bmi1 (Fig 5). In contrast, knockdown of PODXL respectively decreased the IC50 values to 1.4 μM and 2.7 μM, which was completely reversed by overexpression of Bmi1 (Fig 5). The results suggest that PODXL enhances cisplatin chemoresistance in OTSCC cells essentially through Bmi1.

### PODXL reduces cisplatin-induced apoptosis in OTSCC cells through Bmi1

**Fig 6:**
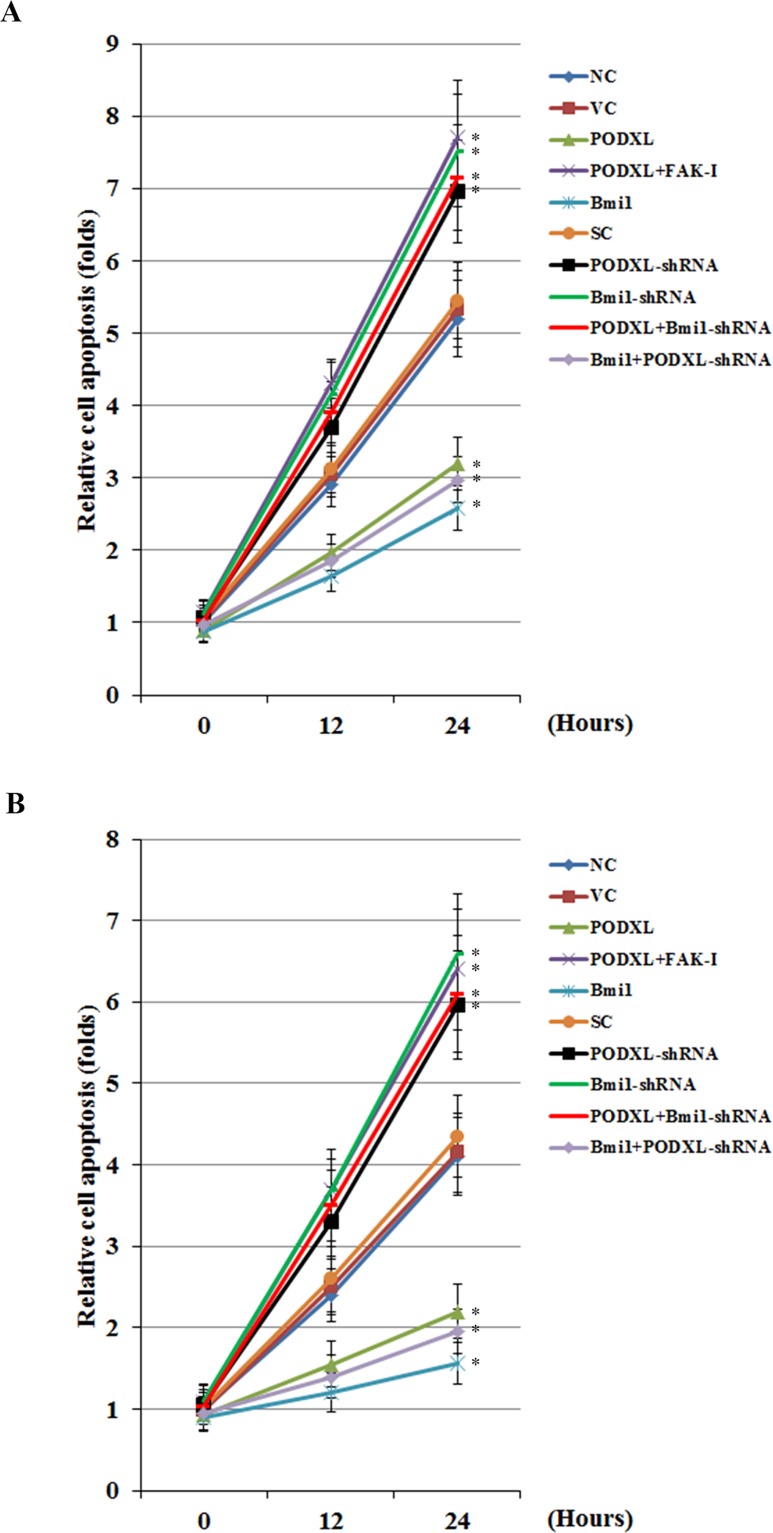
Effect of PODXL/Bmi1 signaling on cisplatin-induced apoptosis in oral tongue squamous cell carcinoma (OTSCC) cells. (A) SCC-4 and (B) Tca8113 OTSCC cells were treated with cisplatin (10 μM) for 12 and 24 hours. Apoptosis was measured with a microplate reader-based TiterTACS in situ apoptosis detection kit (R&D systems) in normal control cells (NC), cells stably transfected with the empty pcDNA 3.1 vector (VC), cells stably transfected with PODXL, cells stably transfected with PODXL and treated with focal adhesion kinase (FAK) inhibitor 14 (50 M) for 24 hours (PODXL+FAK-I), cells stably transfected with Bmi1, cells stably transduced with scramble control shRNA (SC), cells stably transduced with PODXL-shRNA, cells stably transduced with BMI1-shRNA, cells stably transfected with PODXL and transduced with BMI1-shRNA (PODXL+BMI1-shRNA), and cells stably transfected with Bmi1 and transduced with PODXL-shRNA (Bmi1+PODXL-shRNA). Cell apoptosis was shown as fold changes to that of untreated NC (at 0 hour of treatment; designated as 1). *p<0.05 vs. controls (NC, VC and SC).

We next examined the interaction between PODXL and Bmi1 on cisplatin-induced apoptosis in OTSCC cells. As shown in Fig 6, in untreated SCC-4 and Tca8113 cells, overexpression and knockdown of PODXL and Bmi1 showed no significant effect on cell apoptosis compared with the controls. After 24 hours of cisplatin (10 μM) treatment, compared with the controls, overexpression of PODXL significantly decreased cell apoptosis, which was completely reversed by FAK inhibitor 14 or knockdown of Bmi1 (Fig 6). In contrast, knockdown of PODXL markedly increased cell apoptosis, which was completely reversed by overexpression of Bmi1 (Fig 6). The results suggest that PODXL reduces cisplatin-induced apoptosis in OTSCC cells through Bmi1.

### Effect of PODXL/Bmi1 signaling on FAK activity in OTSCC cells

**Fig 7:**
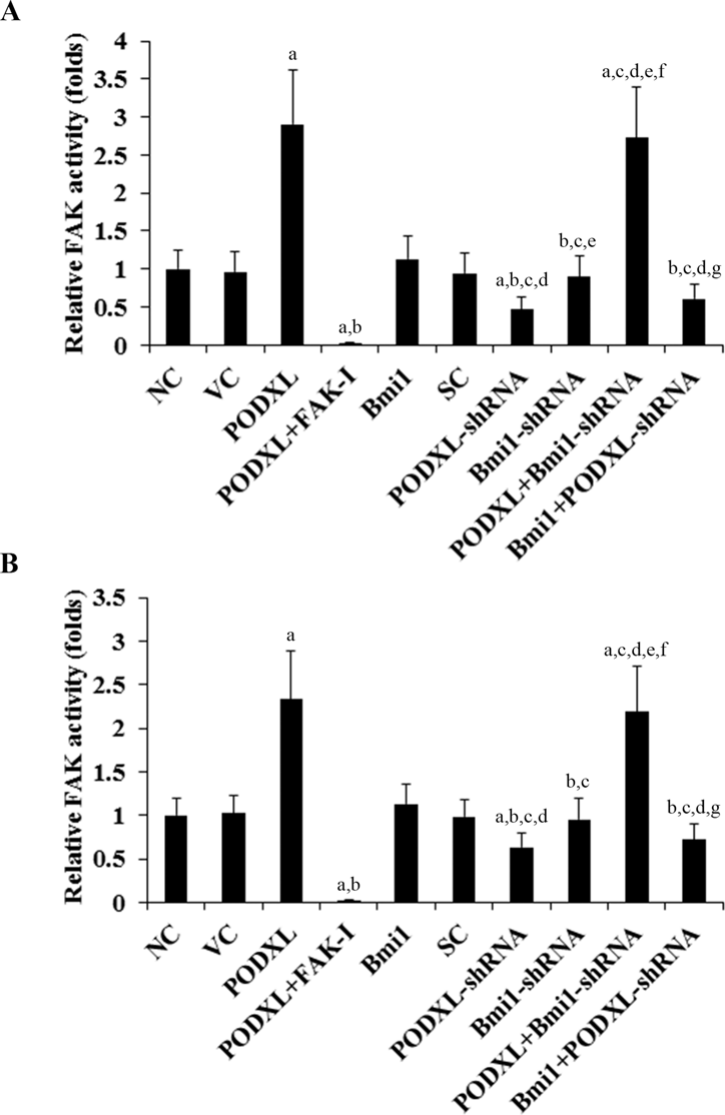
Effect of PODXL/Bmi1 signaling on focal adhesion kinase (FAK) activity in oral tongue squamous cell carcinoma (OTSCC) cells. In (A) SCC-4 and (B) Tca8113 OTSCC cells, the FAK activity was determined with a Universal Tyrosine Kinase Assay kit (Takara Biomedical Technology) in normal control cells (NC), cells stably transfected with the empty pcDNA 3.1 vector (VC), cells stably transfected with PODXL, cells stably transfected with PODXL and treated with focal adhesion kinase (FAK) inhibitor 14 (50 M) for 24 hours (PODXL+FAK-I), cells stably transfected with Bmi1, cells stably transduced with scramble control shRNA (SC), cells stably transduced with PODXL-shRNA, cells stably transduced with BMI1-shRNA, cells stably transfected with PODXL and transduced with BMI1-shRNA (PODXL+BMI1-shRNA), and cells stably transfected with Bmi1 and transduced with PODXL-shRNA (Bmi1+PODXL-shRNA). The FAK activity was shown as fold changes to that of NC (designated as 1). ^a^ *p*<0.05 vs. controls (NC, VC and SC); ^b^ *p*<0.05 vs. PODXL; ^c^ *p*<0.05 vs. PODXL+FAK-I; ^d^ *p*<0.05 vs. Bmi1; ^e^ *p*<0.05 vs. PODXL-shRNA; ^f^ *p*<0.05 vs. Bmi1-shRNA; ^g^ *p*<0.05 vs. PODXL+Bmi1-shRNA.

The above results suggested that PODXL could enhance cisplatin chemoresistance and reduce cisplatin-induced apoptosis in OTSCC cells by up-regulating the expression level of Bmi1 though a FAK-dependent mechanism. Therefore, we next examined the effects of PODXL/Bmi1 signaling on the FAK activity in OTSCC cells. Compared with the controls, overexpression of PODXL respectively induced the FAK activity by 2.9 and 2.3 folds in SCC-4 and Tca8113 cells, which was abolished by FAK inhibitor 14, but not knockdown of Bmi1 (Fig 7). On the other hand, knockdown of PODXL decreased FAK activity by approximately 50% in SCC-4 cells and 40% in Tca8113 cells, which was not significantly altered by overexpression of Bmi1 (Fig 7). The results indicate that PODXL and Bmi1 are respectively upstream and downstream of FAK in the PODXL/Bmi1 signaling axis.

## Discussion

OTSCC is the most common type of OSCC with properties of rapid local invasion and metastatic spread [2]. Innate or acquired resistance to cisplatin, a standard chemotherapy agent for OTSCC, is common in patients with OTSCC [6–8]. Understanding the molecular basis for cisplatin chemoresistance in OTSCC cells may serve as a basis for identification of novel therapeutic targets. A previous study has suggested that PODXL, a transmembrane protein that has been found critical for malignant progression in a variety of cancers [11–18], may also contribute to cancer chemoresistance [18]. Bmi1, a core member of PRC1, has recently been found to induce cell apoptosis and cisplatin chemosensitivity in OTSCC cells [10]. In this study, we demonstrate that PODXL induces cisplatin chemoresistance in OTSCC cells essentially by up-regulating the expression level of Bmi1.

### Crosstalk and functional role of PODXL/FAK/Bmi1 signaling

Overexpression and knockdown of PODXL in OTSCC cells respectively increased and decreased the expression of Bmi1 at both the mRNA and the protein levels. Although our results suggested that PODXL had no direct trans-activating effect on the human Bmi1 promoter in OTSCC cells, we found that PODXL significantly increased the stability of Bmi1, which explains for the PODXL-elevated expression level of Bmi1 in OTSCC cells. In addition, a selective FAK inhibitor readily abolished the PODXL-elevated Bmi1 mRNA level in OTSCC cells without significantly altering the expression of PODXL, suggesting that PODXL increases the stability of Bmi1 mRNA in OTSCC cells through a FAK-dependent mechanism. How PODXL enhances the Bmi1 mRNA stability via FAK in OTSCC cells will be explored in our future studies.

As evidenced by gene overexpression and knockdown experiments, PODXL and Bmi1 individually reduced cisplatin-induced apoptosis and enhanced cisplatin resistance in OTSCC cells. Knockdown of Bmi1 abolished the effect of overexpressing PODXL, while overexpression of Bmi1 completely reversed the effect of knocking down PODXL. The findings indicate that Bmi1 is functionally downstream of PODXL and essentially mediates the enhancing effects of PODXL on cisplatin chemoresistance in OTSCC cells, which corroborates our finding that PODXL up-regulates the expression level of Bmi1 in OTSCC cells.

In agreement with previous reports that PODXL activates FAK [23, 24], this study showed that overexpression and knockdown of PODXL increased and decreased the FAK activity in OTSCC cells, respectively. In contrast, overexpression and knockdown of Bmi1 showed no significant effects on the FAK activity. The results indicate that PODXL and Bmi1 are respectively upstream and downstream of FAK in the PODXL/Bmi1 signaling axis. Previous studies have suggested important roles of FAK in oral carcinogenesis and OTSCC progression [25, 26]. Our findings indicate that FAK mediates the PODXL-elevated expression level of Bmi1 in OTSCC cells, which markedly enhances cisplatin chemoresistance in OTSCC cells. Thus, the importance of FAK in OTSCC progression is at least partially fulfilled through PODXL/Bmi1 signaling.

### Future directions

PODXL reportedly is an essential driver of malignant progression in many cancers [11–18]. Aberrant overexpression of Bmi1 is correlated with advanced stages, aggressive clinicopathological behavior, chemotherapeutic resistance and poor prognosis in a variety of cancers [10]. Therefore, the PODXL/Bmi1 signaling axis identified in this study may also play important roles in the chemoresistance and progression of other cancers besides OTSCC, which needs to be verified in future studies. Cisplatin elicits DNA repair mechanisms by crosslinking DNA [27]. In this study, we only examined the effect of PODXL/Bmi1 signaling on cisplatin chemoresistance. It is unclear whether PODXL/Bmi1 would impact OTSCC cell resistance to other chemotherapeutic components. Further studies with more types of chemotherapy agents would elaborate this issue.

Bmi1 plays a critical role in maintaining the self-renewal capacity of cancer stem cells, which is increasingly recognized to largely account for cancer progression and therapeutic resistance and disease relapse [28, 29]. A recent report has suggested that Bmi1 mediates OTSCC progression through cancer stem cells [30]. PODXL is also a well-known stem cell marker, and is closely related to stem cell marker CD34 and to endoglycan [26]. Thus, based on our findings, it will also be interesting to examine in future studies the role of PODXL/Bmi1 signaling in OTSCC stem-like cells.

### Conclusions

In summary, this study provides the first evidence that PODXL up-regulates the expression level of Bmi1 in OTSCC cells by increasing the stability of Bmi1 mRNA through a FAK-dependent mechanism; this effect leads to enhanced cisplatin chemoresistance in OTSCC cells. This study adds new insights into the molecular mechanisms underlying OTSCC chemoresistance.

## Supporting Information

S1 Fig Titration of dose and duration of actinomycin D treatment in oral tongue squamous cell carcinoma (OTSCC) cells. SCC-4 OTSCC cells were pre-treated with transcription inhibitor actinomycin D for 30 minutes, and then cultured for 1, 2, 3, 4, 5 or 6 hours in medium containing actinomycin D. Different concentrations of actinomycin D were used as follows: 200 μM (0.25 mg/mL), 400 μM (0.5 mg/mL), 800 μM (1 mg/mL), and 1000 μM (1.25 mg/mL). The mRNA level of Bmi1 was determined by real-time RT-PCR assays to measure the inhibition of transcription by actinomycin D, with the mRNA level of Bmi1 at 0 hours (untreated) designated as 100%. Actinomycin D treatment for 1–4 hours at 1 mg/mL was selected based on the titration results.
(TIF)

S2 Fig Transcriptional pulse-chase assays in oral tongue squamous cell carcinoma (OTSCC) cells. The effect of podocalyxin (PODXL) on Bmi1 mRNA stability in (A) SCC-4 and (B) Tca8113 cells was further examined by transcriptional pulse-chase assays using a Click-iT Nascent RNA Capture Kit (Life Technologies). Briefly, the cells were labeled with ethynyl uridine (EU) and incubated at 37°C for 4 hours. Cells were then allowed to recover in EU-free medium for 0, 1, 2 or 4 hours, respectively. Then the labeled RNA was captured and subject to real-time RT-PCR assays to determine the Bmi1 mRNA levels.
(TIF)

S3 Fig IC50 dose-response curves for pcDNA3.1 vector control (VC) and scramble shRNA control (SC) in oral tongue squamous cell carcinoma (OTSCC) cells. The IC50 dose-response curves for VC (*left panel*) and SC (*right panel*) in (A) SCC-4 and (B) Tca8113 OTSCC cells were plotted with GraphPad Prism 5.0 (GraphPad Software).
(TIF)
