# Supplementary figures and images for: Bmi1 Essentially Mediates Podocalyxin-Enhanced Cisplatin Chemoresistance in Oral Tongue Squamous Cell Carcinoma
Source: PLoS One. 2015 Apr 27;10(4):e0123208. doi: 10.1371/journal.pone.0123208 (PMC4411128; doi:10.1371/journal.pone.0123208)

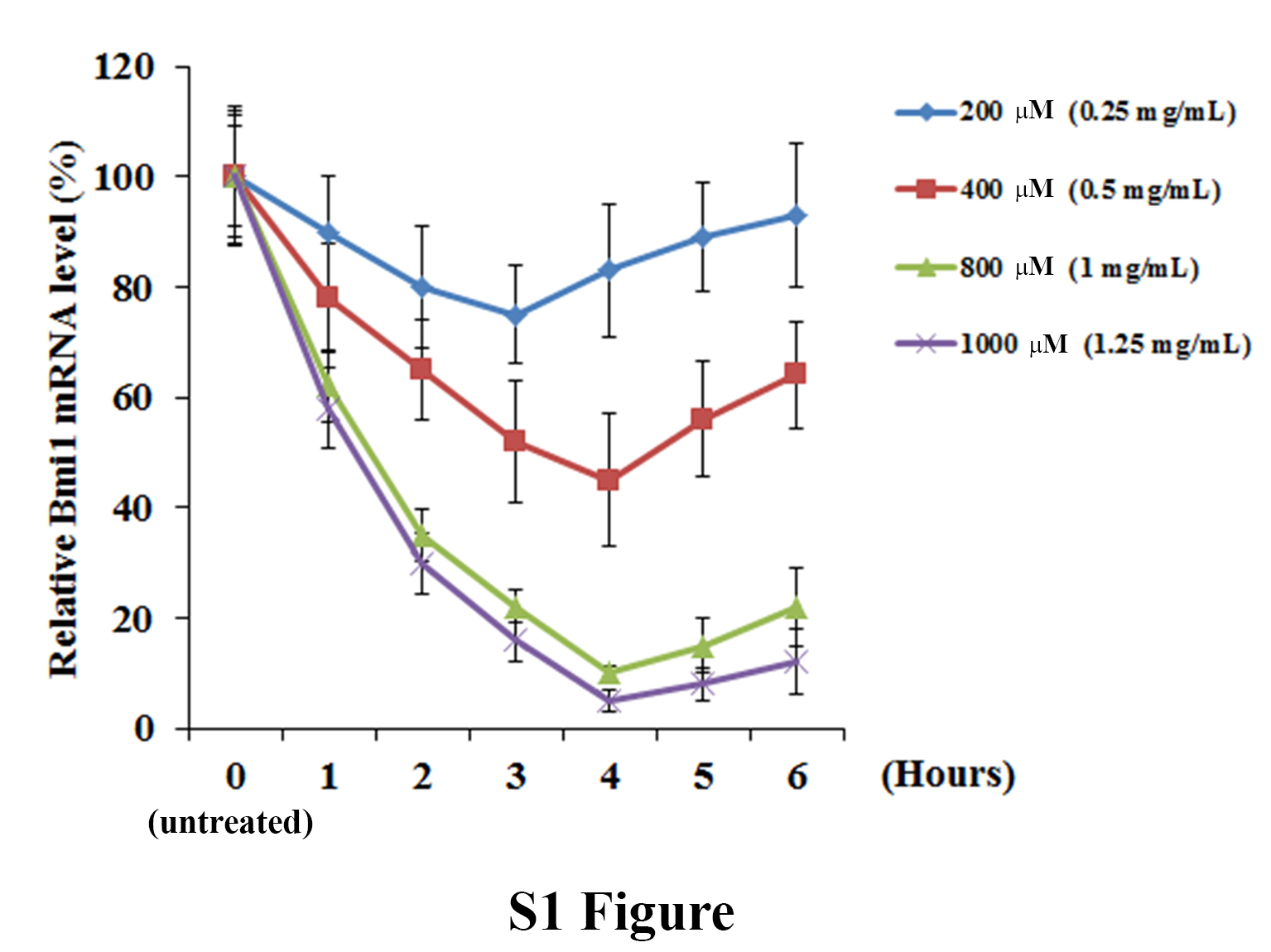

Supplement: S1 Fig — SCC-4 OTSCC cells were pre-treated with transcription inhibitor actinomycin D for 30 minutes, and then cultured for 1, 2, 3, 4, 5 or 6 hours in medium containing actinomycin D. Different concentrations of actinomycin D were used as follows: 200 μM (0.25 mg/mL), 400 μM (0.5 mg/mL), 800 μM (1 mg/mL), and 1000 μM (1.25 mg/mL). The mRNA level of Bmi1 was determined by real-time RT-PCR assays to measure the inhibition of transcription by actinomycin D, with the mRNA level of Bmi1 at 0 hours (untreated) designated as 100%. Actinomycin D treatment for 1–4 hours at 1 mg/mL was selected based on the titration results. (TIF) [file pone.0123208.s001.tif]

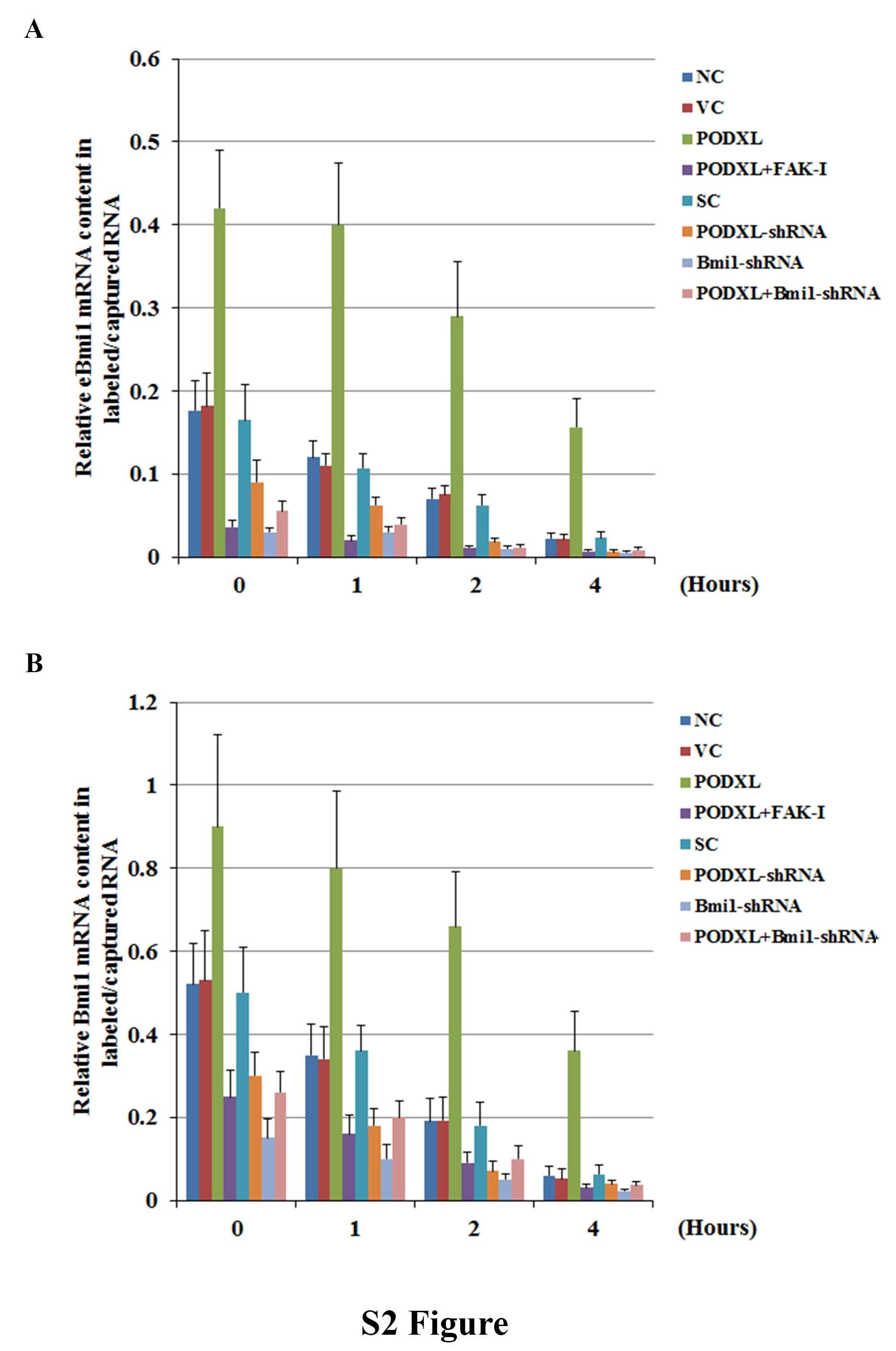

Supplement: S2 Fig — The effect of podocalyxin (PODXL) on Bmi1 mRNA stability in (A) SCC-4 and (B) Tca8113 cells was further examined by transcriptional pulse-chase assays using a Click-iT Nascent RNA Capture Kit (Life Technologies). Briefly, the cells were labeled with ethynyl uridine (EU) and incubated at 37°C for 4 hours. Cells were then allowed to recover in EU-free medium for 0, 1, 2 or 4 hours, respectively. Then the labeled RNA was captured and subject to real-time RT-PCR assays to determine the Bmi1 mRNA levels. (TIF) [file pone.0123208.s002.tif]

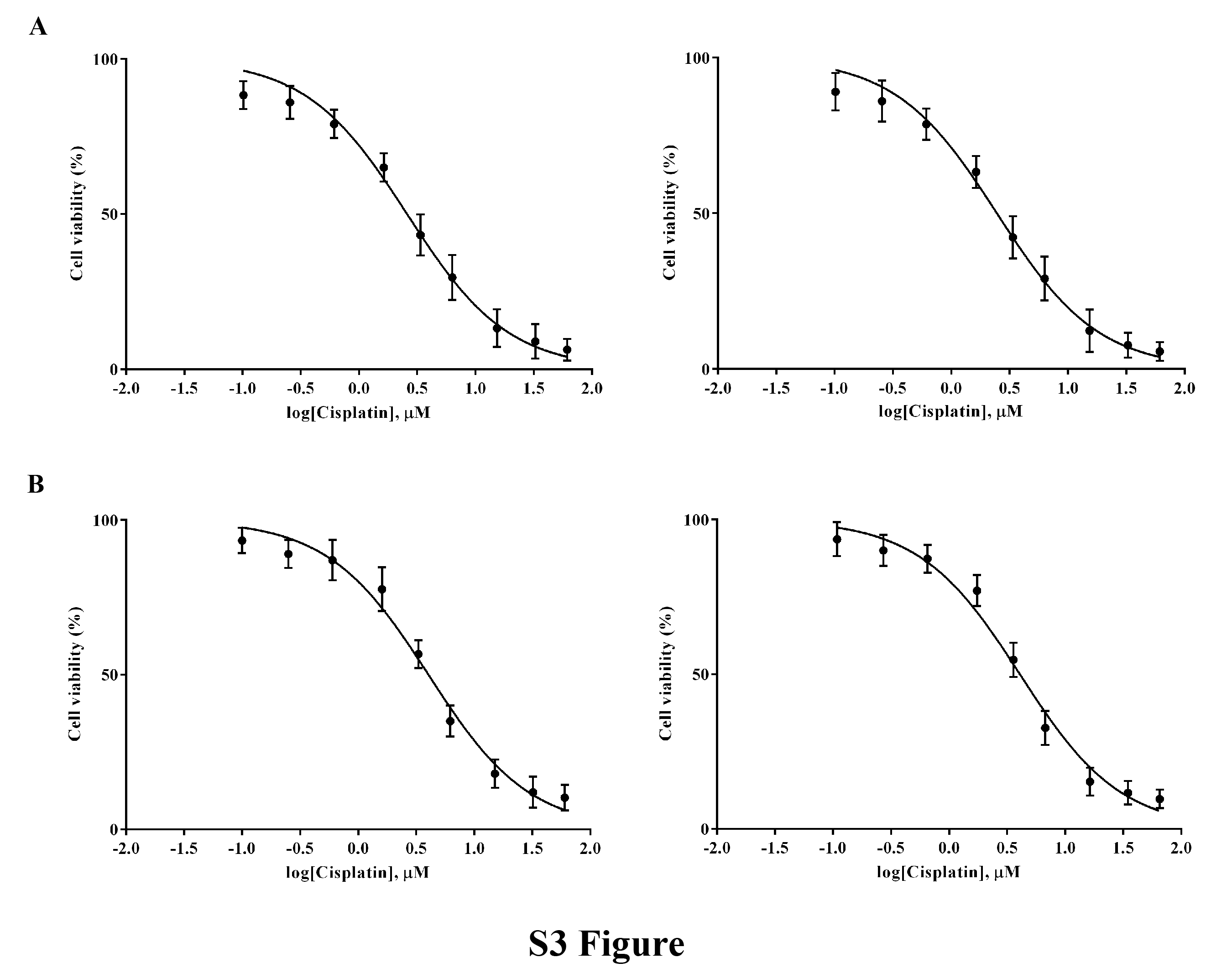

Supplement: S3 Fig — The IC50 dose-response curves for VC (left panel) and SC (right panel) in (A) SCC-4 and (B) Tca8113 OTSCC cells were plotted with GraphPad Prism 5.0 (GraphPad Software). (TIF) [file pone.0123208.s003.tif]
